# Developing novel supersensitive amyloid‐β 43 and pyroglutamate‐modified amyloid‐β 3‐40 assays for Alzheimer’s disease

**DOI:** 10.1002/alz.095753

**Published:** 2025-01-09

**Authors:** Deborah O T Alawode, Amanda J Heslegrave, Henrik Zetterberg

**Affiliations:** ^1^ UK Dementia Research Institute at UCL, London United Kingdom; ^2^ UCL Queen Square Institute of Neurology, London United Kingdom; ^3^ Institute of Neuroscience and Physiology, Sahlgrenska Academy at the University of Gothenburg, Gothenburg Sweden; ^4^ UK Dementia Research Institute, University College London, London United Kingdom; ^5^ UCL Institute of Neurology, Queen Square, London United Kingdom

## Abstract

**Background:**

In light of the poor fold change in the plasma amyloid‐β (Aβ) 42/amyloid‐β 40 ratio (Aβ_42/40_) between Alzheimer’s disease (AD) and control (CTRL) cases, novel Aβ biomarkers are needed which will require harnessing the increased sensitivity offered by a novel modification to the Quanterix Corporation Single molecule array (Simoa) technology.

**Method:**

Simoa assays for the detection of Aβ_43_ and pyroglutamate‐modified Aβ 3‐40 (Aβ_pE3‐40_) have been developed on the standard HD‐X instrument. Aβ_43_ and Aβ_pE3‐40_ were measured in 8 CSF samples (1 AD, 4 mixed phenotype [MP] and 3 CTRL) on the HD‐X instrument, each of which were measured at four dilution levels – neat, 2x, 4x and 8x dilution.

**Result:**

Out of a possible 32 measurements per analyte (four dilution levels per sample), Aβ_43_ was quantifiable in 68.8% of measurements, 72.7% of which fell below the concentration of calibrator B. In three samples, Aβ_43_ was measurable at all four dilution levels, and showed a strong but statistically non‐significant dilution linearity. In contrast, Aβ_pE3‐40_ was quantifiable in 93.8% samples, 96.6% of which fell below the concentrations of calibrator B. In six samples, Aβ_pE3‐40_ was measurable at all four dilution levels. One sample showed a strong but statistically non‐significant dilution linearity, with the remaining five samples showing a poor dilution linearity, suggesting the limits of HD‐X sensitivity had been reached.

**Conclusion:**

The increased sensitivity provided by the modification to the Simoa SRx is needed to accurately quantify Aβ_43_ and Aβ_pE3‐40_ in CSF and subsequently plasma. Work is ongoing to transfer these novel assays from the HDx onto the modified SRx instrument.

